# Central Roles of ZmNAC128 and ZmNAC130 in Nutrient Uptake and Storage during Maize Grain Filling

**DOI:** 10.3390/genes15060663

**Published:** 2024-05-23

**Authors:** Di Peng, Shuxing Pan, Xin Du, Erwang Chen, Junjun He, Zhiyong Zhang

**Affiliations:** 1 School of Life Sciences, Division of Life Sciences and Medicine, University of Science and Technology of China, Hefei 230027, China; dipeng@mail.ustc.edu.cn (D.P.); shuxingpan@mail.ustc.edu.cn (S.P.); duxin2023@ustc.edu.cn (X.D.); ewchen@ncgr.ac.cn (E.C.); 2 South Subtropical Crop Research Institute, Chinese Academy of Tropical Agricultural Science, Zhanjiang 524091, China; hbj46@163.com

**Keywords:** maize grain, gene regulation, transporter, nutrient uptake, storage metabolism

## Abstract

Grain filling is critical for determining yield and quality, raising the question of whether central coordinators exist to facilitate the uptake and storage of various substances from maternal to filial tissues. The duplicate NAC transcription factors ZmNAC128 and ZmNAC130 could potentially serve as central coordinators. By analyzing differentially expressed genes from *zmnac128 zmnac130* mutants across different genetic backgrounds and growing years, we identified 243 highly and differentially expressed genes (hdEGs) as the core target genes. These 243 hdEGs were associated with storage metabolism and transporters. ZmNAC128 and ZmNAC130 play vital roles in storage metabolism, and this study revealed two additional starch metabolism-related genes, *sugary enhancer1* and *hexokinase1*, as their direct targets. A key finding of this study was the inclusion of 17 transporter genes within the 243 hdEGs, with significant alterations in the levels of more than 10 elements/substances in mutant kernels. Among them, six out of the nine upregulated transporter genes were linked to the transport of heavy metals and metalloids (HMMs), which was consistent with the enrichment of cadmium, lead, and arsenic observed in mutant kernels. Interestingly, the levels of Mg and Zn, minerals important to biofortification efforts, were reduced in mutant kernels. In addition to their direct involvement in sugar transport, ZmNAC128 and ZmNAC130 also activate the expression of the endosperm-preferential nitrogen and phosphate transporters *ZmNPF1.1* and *ZmPHO1;2*. This coordinated regulation limits the intake of HMMs, enhances biofortification, and facilitates the uptake and storage of essential nutrients.

## 1. Introduction

Grain filling encompasses intricate biochemical and physiological processes, including the uptake of macro- and micronutrients from maternal tissues to developing grains, along with the synthesis of storage compounds within endosperms or embryos. This phase is pivotal in determining grain yield and quality. The relatively large caryopsis of maize (*Zea mays*), combined with its agricultural and scientific importance, positions it as an optimal system for investigating seed development in the grass family [1].

Maize plants have developed a highly specialized maternal/filial interface structure in the basal kernel zone, consisting of the basal endosperm transfer layer (BETL), placenta-chalazal (PC), and pedicel (PED), to facilitate nutrient transport from the maternal plant to the developing endosperm [2]. To date, four sugar transporters (*ZmSWEET4c* [sugars will eventually be exported transporter 4c] [3], *ZmSUGCAR1* [sucrose and glucose carrier 1] [4], *ZmSUT1* [sucrose transporter 1] [5], *ZmSUT7* [6]) and one cell wall invertase-encoding gene (*ZmMN1* [miniature 1]) [7], in the BETL, have been shown to function in the uptake of carbohydrates/carbons (C). Recently, duplicate maize PHO1-type phosphate (P) transporters, *ZmPHO1;2a* and *ZmPHO1;2b* have been shown to function in P reallocation and grain filling [8,9]. Additionally, the Zn-NA (nicotianamine) transporter gene *ZmYSL2* has been found to be involved in the zinc (Zn) upload to maize kernels [10]. Although progress has been made in understanding the uptake of C, P, and Zn in maize grains, the uptake of other essential nutrients such as nitrogen (N) and magnesium (Mg) has not been fully elucidated.

A second critical aspect of the grain-filling stage is the conversion of nutrients into seed-storage compounds such as starch, protein, and lipids. In grains, C is mainly stored in the form of starch within endosperm starch granules, which accounts for approximately 70% of the kernel weight [1]. While sugar metabolism is complex, the starch synthesis pathway in the maize endosperm is well understood. The key enzymes involved in starch synthesis in the maize endosperm include the following: sucrose synthases (such as shrunken1 [Sh1], SUS1, and SUS2); UDP-glucose pyrophosphorylase; ADP-glucose pyrophosphorylase (AGPase), which is composed of a small subunit (brittle2 [Bt2]) and a large subunit (shrunken2 [Sh2]); ADP-glucose (ADPG) transporter (brittle1 [Bt1]); soluble starch synthases (SS-I, -II, -III, -IV, and -V); granule-bound starch synthase (GBSS1, or waxy [Wx]); starch-branching enzymes (such as SBE-IIa and -IIb); and starch-debranching enzymes (maize pullulanase1 [Zpu1]) [11]. In terms of N, the maize seed storage proteins are primarily composed of endosperm prolamins (zeins) and embryo globulins. The α-, β-, γ-, and δ-zeins account for approximately 60% of the total seed proteins [12]. The abundance of zeins largely determines the amino acid composition of kernels, but their low lysine and tryptophan contents negatively impact protein quality [12].

During the filling stage, major starch metabolism genes and zeins are highly and specifically expressed in the endosperm [6,13], highlighting the essential role of transcriptional regulation in coordinating this complex process. Multiple transcription factors (TFs), including opaque2 (O2) [14,15,16,17], prolamin-box binding factor 1 (PBF1) [18,19], O2 heterodimerizing proteins (OHP1 and OHP2) [20,21], duplicate indeterminate domain (IDD) TFs (NKD1 and NKD2) [22], one MADS-type TF (ZmMADS47) [23], one bZIP-type TF (ZmbZIP22) [24], and duplicate NAC-type TFs (ZmNAC128 and ZmNAC130) [25,26], have been shown to directly regulate zein gene expression. Additionally, O2, PBF1, NKD1, NKD2, ZmNAC128, ZmNAC130, and opaque11 (O11) have been identified as regulators of starch metabolism genes [22,25,26,27,28]. Recently, ABSCISIC ACID INSENSITIVE 19 (ZmABI19) was shown to directly regulate multiple important TFs, such as O2, PBF1, ZmbZIP22, ZmNAC130, and O11, in the endosperm [29]. Furthermore, ZmABI19 interacts with the basic leucine zipper 29 to synergistically regulate *O2* expression [30]. O2, serving as a core TF in maize endosperm filling [14,15,16,17], can genetically or molecularly interact with ZmNAC128/ZmNAC130, or NKD1/NKD2 to effectively coordinate endosperm filling [26,31]. Recently, two genes downstream of O2, namely, the GRAS domain-containing protein ZmGRAS11 [32] and the RNA polymerase common subunit ZmRPABC5b [33], have been characterized to play essential roles in endosperm filling development. However, the regulatory mechanisms underlying nutrient entry into grains are poorly understood, despite advancements in our understanding of nutrient storage metabolism.

ZmNAC128 and ZmNAC130 have emerged as key players in maize kernel development, as their mutations result in severe defects in grain filling, such as significant reductions in starch and protein content in mature kernels [25,26]. These two NACs not only act as core regulators of starch and zein synthesis but also participate in the regulation of sugar and zinc uptake. These intriguing findings prompted us to explore the existence of central regulators that coordinate both the uptake and storage of macro- and micronutrients from mother plants into filial grains. Therefore, it is imperative to investigate whether ZmNAC128 and ZmNAC130 fulfill this role as central regulators. This approach holds great potential for uncovering essential grain-filling mechanisms, which can ultimately contribute to crop yield and quality improvements.

## 2. Materials and Methods

### 2.1. Plant Materials and Growth Conditions

The knockout lines of ZmNAC128 and ZmNAC130 in the KN5585 inbred background were previously generated [26]. This study utilized three sets of *zmnac128 zmnac130* mutant materials, with the first and second sets derived from our previous study [26], and the third set specifically developed for this study. The *zmnac128 zmnac130* mutant and the corresponding wild type in the B73 × KN5585 background were generated from the F_1_ cobs by crossing the inbred B73 with *zmnac128 zmnac130* in the KN5585. The F_1_ plants from the cross of B73 with KN5585 *zmnac128 zmnac130* were grown in the field. We selected self-pollinated cobs from these T_1_ plants to complete the subsequent experiments. For this study, endosperms of the wild type (WT) and *zmnac128 zmnac130* mutant were genotyped and collected from the F1 cobs that had been pollinated at the same time. All maize materials were planted in a field located in Hefei (Anhui Province, China) from March to July in both 2022 and 2023. Given the complexity of the genetic background of B73 × KN5585, we used the *zmnac128 zmnac130* mutant in the KN5585 background, as well as the KN5585 (wild type) itself, as the samples for all subsequent molecular experiments and various substances and element analysis experiments in this study.

### 2.2. RNA Sequencing (RNA-Seq)

The RNA-seq method employed in this study followed the same procedure outlined in our previous work [26]. Total RNA was extracted from more than three 16-days-after-pollination (16-DAP) endosperms from the WT and *zmnac128 zmnac130* mutant using TRIzol reagent (Invitrogen, Carlsbad, CA, USA). Subsequently, libraries were prepared and sequenced on a DNBSEQ-T7 platform, generating 150 bp paired-end reads. The resulting clean reads were aligned to the B73 reference genome (RefGen_v5) using the HISAT2 program [34]. A differential expression analysis was performed using the R package DESeq [35], with differentially expressed genes defined as those exhibiting an absolute log2 fold change (FC) greater than 1 and an adjusted *p*-value below 0.05.

### 2.3. Quantification of Lipid Content in Mature Kernels

More than ten mature kernels were milled into flour and subsequently filtered using a 50-mesh stainless steel screen. The lipid content of the resulting milled flour was determined using a pre-constructed calibration model based on an MPA-type Fourier-transform near-infrared spectrophotometer (Bruker, Ettlingen, Germany). Sample spectra were collected on the MPA spectrophotometer, and the modeling algorithm employed was a partial least squares regression. Each type of mature kernel flour was divided into four portions for measurement purposes.

### 2.4. Analysis of Element Content in Mature Kernels

The element profiling was conducted using inductively coupled plasma–mass spectrometry (ICP-MS), following previously established methods [10]. For each sample, six dry mature kernels were ground into powder, and 2–5 mg of each sample was collected for the elemental profiling. To ensure consistency, all samples were normalized using a heuristic algorithm that utilized the best-measured elements, as described previously [36].

### 2.5. Determination of Phytohormone Content in 16-DAP Endosperm

Approximately 100 mg of frozen 16-DAP endosperm powder was extracted in 1 mL of ice-cold 50% aqueous ACN (*v*/*v*). The samples were sonicated for 3 min at 4 °C and subsequently extracted using a benchtop laboratory rotator for 30 min at 4 °C. After centrifugation (10 min, 10,000× *g*, 4 °C), the supernatant was transferred to clean plastic microtubes. All samples were purified using C18 reversed-phase, polymer-based, solid-phase extraction (RP-SPE) cartridges, that had been washed with 1 mL of MeOH and 1 mL of sterile deionized water, then equilibrated with 50% aqueous ACN (*v*/*v*). After loading a sample, the cartridge was then rinsed with 1 mL of 30% ACN (*v*/*v*) and this fraction was collected. After this single-step SPE, the samples were evaporated until dry under a gentle stream of nitrogen and stored at −20 °C until analysis. For UHPLC–ESI–MS/MS analysis, the samples were dissolved in 200 μg/mL of 30% ACN (*v*/*v*) and transferred to insert-equipped vials.

The sample extracts were analyzed using a UPLC-Orbitrap-MS system (UPLC, Vanquish, Thermo Fisher Scientific, Carlsbad, CA, USA). The HRMS data were recorded on a Q Exactive hybrid Q-Orbitrap mass spectrometer equipped with a heated ESI source (Thermo Fisher Scientific, Carlsbad, CA, USA) utilizing the SIM MS acquisition methods. Data were acquired on the Q-Exactive using Xcalibur 4.1 (Thermo Fisher Scientific, Carlsbad, CA, USA) and processed using TraceFinder™4.1 Clinical (Thermo Fisher Scientific, Carlsbad, CA, USA).

### 2.6. RNA Extraction and Quantitative RT-PCR

The total RNA was extracted from maize endosperms at 12- and 20-DAP using TRIzol reagent (Invitrogen). Subsequently, 2 µg of total RNA was utilized to synthesize the first-strand cDNA with the HiScript^®^ II 1st Strand cDNA Synthesis Kit (R212, Vazyme, Nanjing, China). Using SYBR Green (Genstar, Beijing, China), the quantitative RT-PCR assay was performed on a CFX Connect real-time PCR system (Bio-Rad, Hercules, CA, USA) following the standard operating manual. We used the default 100% efficiency setting on the CFX Connect real-time PCR system.

To estimate the relative expression of the target gene between the WT and mutants, the comparative CT (ΔΔCT) method [37] was used with *ZmActin* serving as the reference. The primer sequences are listed in Table S3.

### 2.7. Dual-Luciferase Reporter (DLR) Assay

Leaves prepared for maize protoplasts were collected from more than 20 two-week seedlings of the inbred B73 cultivated in darkness. The process we followed for the DLR assay was in accordance with the method described previously [26]. The protoplast isolation involved the process of zymolysis using Cellulose R10 (Yakult, Tokyo, Japan) and Macerozyme R10 (Yakult). The recombinant vector pRI101 (Clontech, Takara Bio USA, Inc., San Jose, CA, USA) and pGreenII 0800-LUC were transfected into a protoplast using polyethylene glycol (PEG)-calcium. The protoplast was cultivated overnight for the further detection of the LUC/REN ratio using a dual-luciferase reporter assay system (Promega, Madison, WI, USA). The vector pRI101 (Clontech, Takara Bio USA, Inc., San Jose, CA, USA) was used for the expression of *ZmNAC128* and *ZmNAC130* under the control of the 35S promoter and the vector pGreenII 0800-LUC was used to generate the reporter constructs by cloning the promoters of different target genes upstream of LUC. The primers are listed in Table S3.

### 2.8. Phylogenetic Analysis

The protein sequence was aligned and analyzed by ClustalW with the MEGA11 software. The neighbor-joining tree was constructed with the MEGA11 software (http://megasoftware.net/, accessed on 24 June 2022) according to the protein sequence alignment.

### 2.9. Statistical Analysis

Data processing of means, standard deviations, and p-values was performed with Microsoft Excel (2019) using AVERAGE, STDEV.S, and Student’s *t*-test, respectively. Statistical tests involving a one-way ANOVA were performed with GraphPad Prism (version 8.4.3). The hierarchical clustering analysis using Pearson correlation and visualization was performed with the corrplot package (v 0.92). The raw data and detailed statistical analysis are in Dataset S2.

## 3. Results

### 3.1. Generation and Analysis of Transcriptomes from Endosperms of zmnac128 zmnac130 Mutants across Different Genetic Backgrounds and Growing Years

Our previous studies revealed that mutations in *ZmNAC128* and *ZmNAC130*, which are specifically and highly expressed duplicate TFs in endosperm filling, result in severe defects in kernel filling [25,26]. We identified more than 10 genes as direct targets, which partly explains the role of *ZmNAC128* and *ZmNAC130* in endosperm filling, but these genes account for only a small fraction of the thousands of genes affected by the loss-of-function mutation of these two NAC TFs. Therefore, an essential question arises: how does their core regulatory network operate?

To explore the core genes in the regulatory network, we obtained three sets of transcriptomes from the endosperms of the *zmnac128 zmnac130* mutants and the corresponding wild-type (WT) or nontransgenic (NT) siblings under different genetic backgrounds and growing years at 16 DAP. Two sets of data were generated in our previous study [26]: one from the KN5585 genetic background (referred to as “set-1”) and the other from the KN5585 × B73 background (referred to as “set-2”) grown on a farm in Hefei City, China, during the summer of 2022. Set-3 was generated on the same farm during the summer of 2023, also using the KN5585 × B73 background. Among all 14 transcriptomes from these three experiments, an average of 43,560,203 (92.93%) clean reads were uniquely mapped to the maize B73 reference genome (Zm-B73-REFERENCE-NAM-5.0) (Table S1). The fragments per kilobase of the exon model per million mapped fragments (FPKM) were used to quantify gene expression.

**Figure 1:**
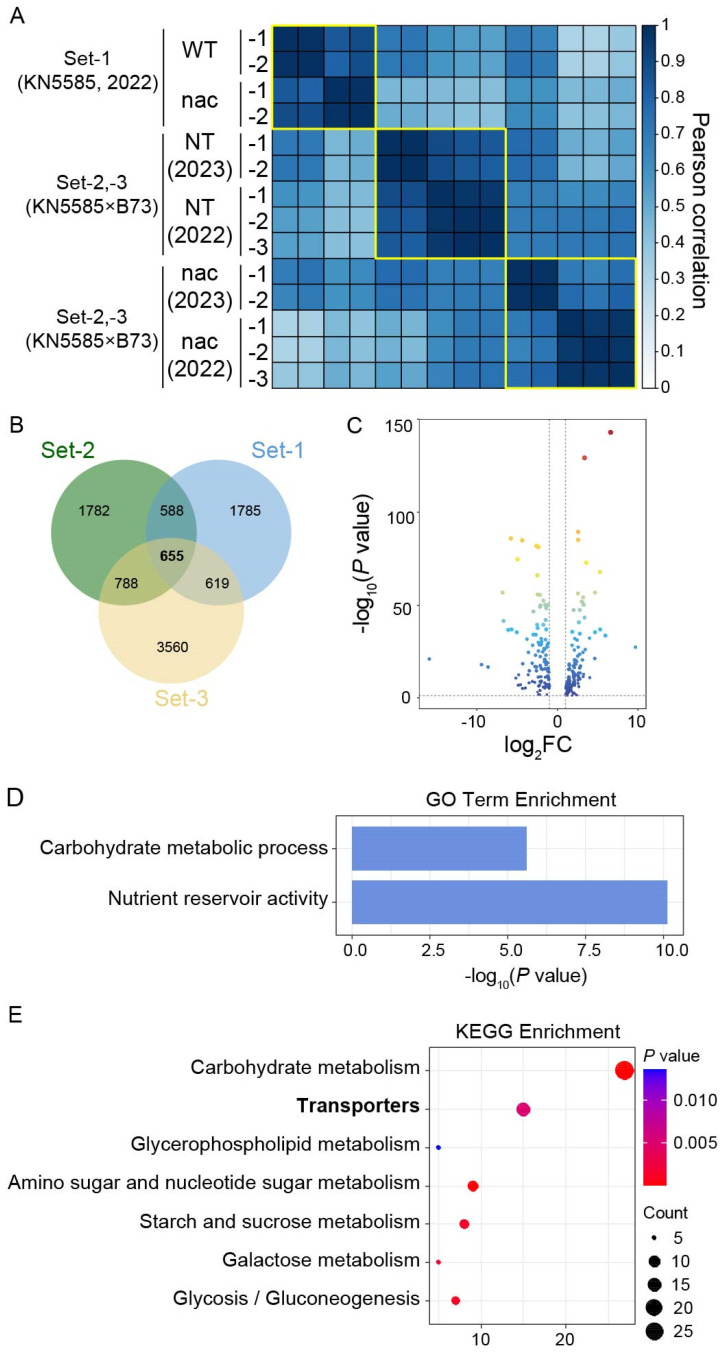
An analysis of transcriptomes from 16-DAP endosperms of *zmnac128 zmnac130* mutants with different genetic backgrounds and growing years. (**A**) A hierarchical clustering analysis of three sets of transcriptomes based on a Pearson correlation of gene expression. WT, wild type. NT, nontransgenic sibling segregated from crossing the inbred B73 with the *zmnac128 zmnac130* mutant in the inbred KN5585. nac, *zmnac128 zmnac130* mutant. -1, -2, and -3 represent three independent samples. 2022 and 2023 represent the two growing years. (**B**) A Venn diagram shows the dEGs in the three sets of transcriptomes. The number in each circle represents the number of dEGs. (**C**) A volcano plot displays the downregulated and upregulated genes among the hdEGs. (**D**,**E**) Gene Ontology (GO) and Kyoto Encyclopedia of Genes and Genomes (KEGG) enrichment analyses of the 243 hdEGs are shown in (**C**).

To evaluate the influence of genetic background and growing years on the transcriptome, we performed hierarchical clustering analysis using a Pearson correlation of gene expression among samples. The results revealed a distinct segregation between transcriptomes of the two genetic backgrounds, as well as a noticeable separation of transcriptomes from the same genetic background across two growing years (Figure 1A). Two possible explanations for these results are as follows: (1) The hybrid progeny of KN5585 and B73 introduced increased complexity in the transcriptomes of set-2 and set-3. (2) The genetic background had a stronger impact on transcriptome variation than growing years. However, further research is needed to test these hypotheses. Identifying highly and differentially expressed genes (hdEGs) under different genetic backgrounds and growing years will help to unravel the core regulatory mechanism of ZmNAC128 and ZmNAC130 with reduced interference.

### 3.2. Identifying the hdEGs across Different Genetic Backgrounds and Growing Years as Core Members in the Regulatory Network of ZmNAC128 and ZmNAC130

Using a threshold of log_2_(fold change, FC) > 1 and *p* value < 0.05, we identified 3813, 3647, and 5622 differentially expressed genes (dEGs) in the transcriptomes of set-1, set-2, and set-3, respectively. A Venn diagram showed that 655 dEGs were shared among all three sets (Figure 1B). We further screened highly expressed genes with FPKM values > 10 among the 655 dEGs based on the data from the KN5585 background. This led to the identification of 243 hdEGs, including 130 upregulated genes with FPKM values > 10 in the *zmnac128 zmnac130* mutant and 113 downregulated genes with FPKM values > 10 in the WT (Figure 1C; Dataset S1). These 243 hdEGs were considered core targets of ZmNAC128 and ZmNAC130 and were further investigated in this study.

Gene Ontology (GO) analysis of these 243 hdEGs revealed significant enrichment in two terms: ‘carbohydrate metabolic process’ and ‘nutrient reservoir activity’ (Figure 1D). These results support our previous findings on the regulatory roles of these genes in starch and zein synthesis. Interestingly, the Kyoto Encyclopedia of Genes and Genomes (KEGG) analysis also indicated that transporters were significantly enriched in these 243 hdEGs (Figure 1E). The results suggested that the core role of ZmNAC128 and ZmNAC130 was in nutrient uptake and storage. Our previous studies revealed the important roles of these TFs in the uptake of sugars and Zn, but further investigation is needed to determine the regulatory mechanisms involved in the transport of other nutrients and elements.

### 3.3. Identifying Starch Metabolism-Related Sugary Enhancer 1 (SE1) and Hexokinase 1 (HXK1) as Direct Targets of ZmNAC128 and ZmNAC130

Following our previous findings [25,26], we observed an enrichment of storage metabolism associated with carbon (C), nitrogen (N), and phosphate (P) in these 243 hdEGs (Figure 1D,E). This finding confirmed that storage metabolism is central to the regulatory network of ZmNAC128 and ZmNAC130.

**Figure 2:**
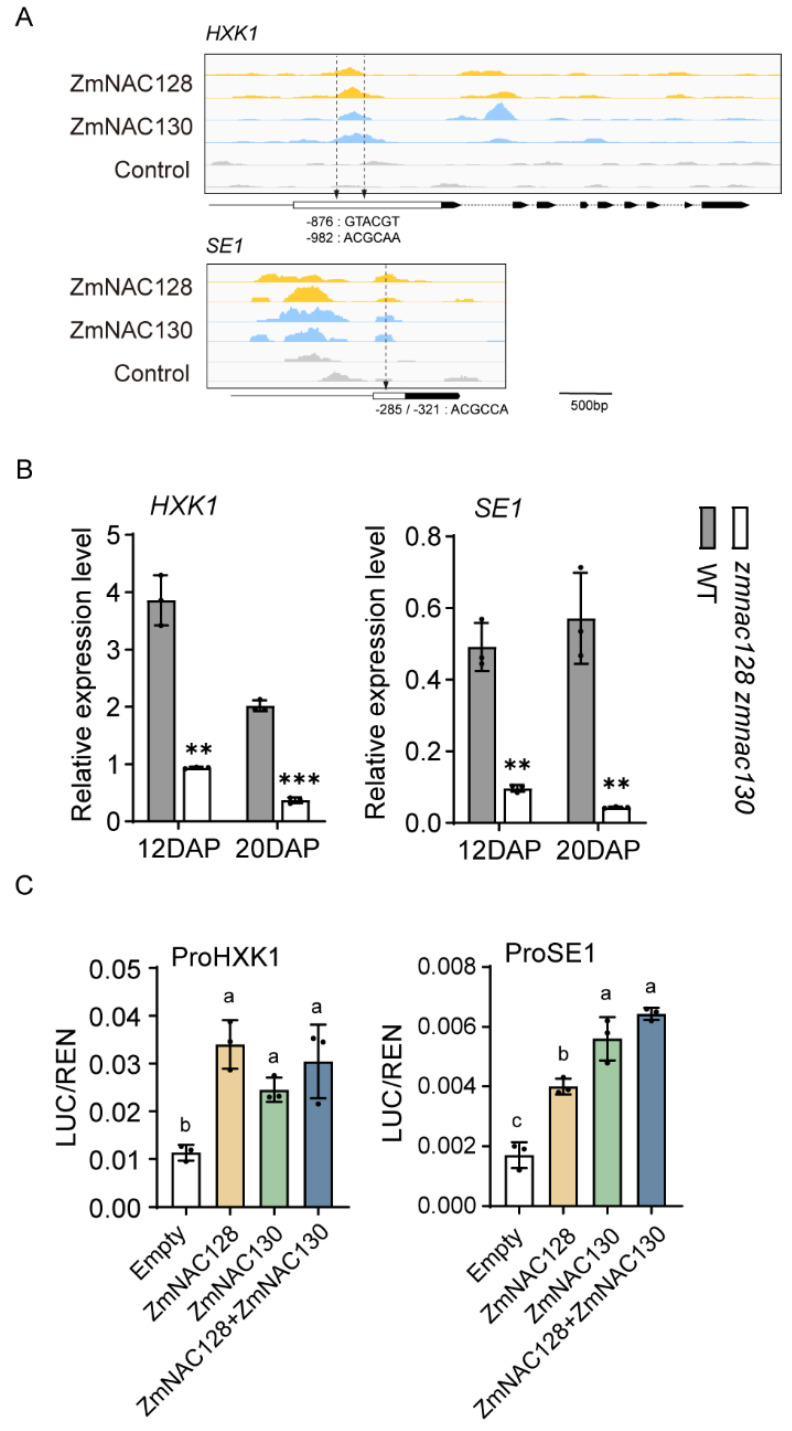
The identification of two carbohydrate metabolism-related genes, *HXK1* and *SE1*, as direct targets of ZmNAC128 and ZmNAC130. (**A**) DAP-seq analysis shows the binding peaks of ZmNAC128 and ZmNAC130 on these two gene promoters. The cis-elements (‘ACGCAA’ and ‘GTACGT’), which were previously identified [25,26], are marked by arrows in the promoters. (**B**) A real-time PCR indicates the relative expression levels of these genes in 12- and 20-DAP endosperms of the *zmnac128 zmnac130* mutant compared to those of the WT. The *ZmActin* was used as a reference gene for normalization, employing the default 100% efficiency setting for detected genes on the CFX Connect real-time PCR system. (**C**) DLR assays detect LUC activities driven by the promoters in the presence of ZmNAC128, ZmNAC130, or both. Empty, the negative control. The data represent the mean ± standard deviation (SD) of three independent samples (**B**,**C**). Significant differences (** *p* < 0.01 and *** *p* < 0.001) were determined using a Student’s *t*-test (**B**). Different lowercase letters indicate significant differences according to a one-way ANOVA with Tukey’s multiple comparisons test (*p* < 0.05) (**C**).

Our previous studies demonstrated that ZmNAC128 and ZmNAC130 coordinate sugar uptake and starch metabolism during endosperm filling [25,26]. ZmNAC128 and ZmNAC130 directly regulate the expression of two crucial sugar transporter genes, *ZmSWEET4c* and *ZmSUGCAR1*, with *ZmSWEET4c* being one of the 243 hdEGs. Among the 243 hdEGs, we found two downregulated genes, *SE1* (Zm00001eb115450) and *HXK1* (Zm00001eb121400), which are related to endosperm starch metabolism (Dataset S1) [38,39]. A real-time PCR confirmed that the expression of the *SE1* and *HXK1* genes was also significantly downregulated in the 12- and 20-DAP endosperms of the *zmnac128 zmnac130* mutant (Figure 2B). By analyzing the DNA affinity purification sequencing (DAP-Seq) data for ZmNAC128 and ZmNAC130 which were previously produced [26], we detected whether there were distinct binding peaks for two NACs in the *SE1* and *HXK1* promoters. The results indicated that there were distinct peaks bound by ZmNAC128 and ZmNAC130 within the two promoters with conserved NAC-binding motifs (Figure 2A). A dual-luciferase reporter (DLR) assay was further performed to detect the transactivation activities of the two promoters. The results indicated that, compared to that of the empty control, the luciferase (LUC) activity driven by the *SE1* or *HXK1* promoter was significantly increased by ZmNAC128 or ZmNAC130, but the two NACs together did not further enhance the LUC activity (Figure 2C). This finding is consistent with our previous findings that ZmNAC128 and ZmNAC130 have functional redundancy [25,26]. Thus, combined with our previous findings, up to eight starch metabolism-related genes are directly regulated by ZmNAC128 and ZmNAC130. These findings highlight the vital role of ZmNAC128 and ZmNAC130 in controlling sugar uptake and storage in filling endosperms.

### 3.4. Regulatory Roles of ZmNAC128 and ZmNAC130 on Toxic HMM Accumulation and Biofortification in Maize Kernels

**Figure 3:**
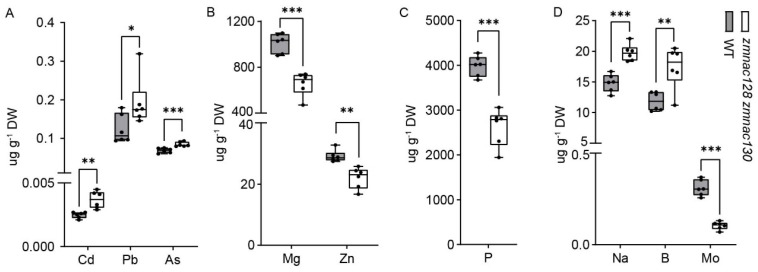
ZmNAC128 and ZmNAC130 affect the accumulation of numerous elements in mature kernels. (**A**) The levels of three toxic HMMs were significantly increased in mature kernels of the *zmnac128 zmnac130* mutant. (**B**) The levels of two biofortified elements were significantly decreased in mature kernels of the *zmnac128 zmnac130* mutant. The Zn content data were from our previous publication [25,26]. (**C**) The P level was significantly decreased in mature kernels of the *zmnac128 zmnac130* mutant. (**D**) The levels of Na, B, and Mo were significantly changed in mature kernels of the *zmnac128 zmnac130* mutant. The data represent the mean ± SD of 5 independent samples (**A**–**D**). Significant differences (* *p* < 0.05, ** *p* < 0.01, and *** *p* < 0.001) were determined via Student’s *t*-test.

The enrichment of ‘transporters’ in the 243 hdEGs led us to investigate their relationship with ZmNAC128 and ZmNAC130. This category included nine upregulated and eight downregulated transporter genes (Table 1). Among the nine upregulated transporters, six were closely associated with heavy-metal and metalloid (HMM) transport [40,41,42], including one C-type ABC transporter/multidrug resistance protein-related transporter ZmMRPA14, one natural resistance-associated macrophage protein ZmNRAMP5, and four multidrug and toxic compound extrusion transporters (ZmMATE4, ZmMATE11, ZmMATE17, and ZmMATE48). Therefore, we utilized inductively coupled plasma-mass spectrometry (ICP-MS) to measure the levels of various elements in mature kernels of the *zmnac128 zmnac130* mutant. In addition to Zn [26], the levels of eight other elements were also significantly altered in *zmnac128 zmnac130* kernels, including cadmium (Cd), lead (Pb), arsenic (As), boron (B), natrium (Na), phosphorus (P), magnesium (Mg), and molybdenum (Mo) (Figure 3A–D).

**Figure 4:**
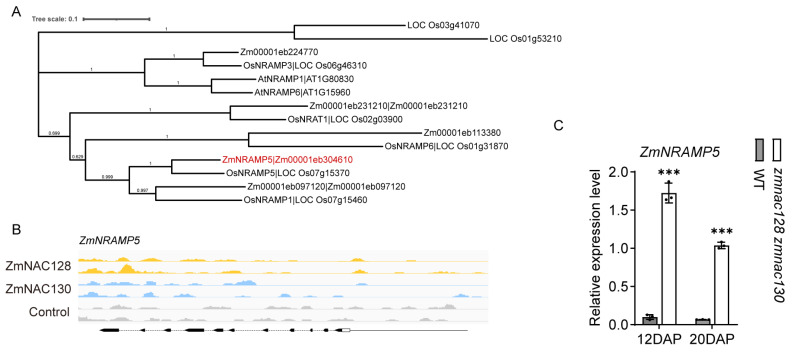
The identification of *ZmNRAMP5* as the direct target gene of ZmNAC128 and ZmNAC130. (**A**) A phylogenetic analysis depicting the relationship among NRAMP proteins from maize, rice, and Arabidopsis. The tree was generated by MEGA11 software using the blastp result of NRAMP protein sequences. Blastp is performed by phytozome v13 and the N-J method is used with 1000 bootstrap. (**B**) DAP-seq analysis shows the binding peaks of ZmNAC128 and ZmNAC130 on the promoter of *ZmNRAMP5*. (**B**) A real-time PCR indicating the relative expression levels of *ZmNRAMP5* in 12- and 20-DAP endosperms of the *zmnac128 zmnac130* mutant compared to those of the WT. *ZmActin* was used as a reference gene for normalization. (**C**) DLR assays detecting LUC activities driven by the *ZmNRAMP5* promoter in the presence of ZmNAC128, ZmNAC130, or both. Empty, the negative control. The data represent the mean ± SD of 3 independent samples (**B**,**C**). Significant differences (*** *p* < 0.001) were determined using a Student’s *t*-test (**B**). Different lowercase letters indicate significant differences according to a one-way ANOVA with Tukey’s multiple comparisons test (*p* < 0.05) (**C**).

Notably, Cd, Pb, and As, which act as toxic HMMs closely related to grain safety [43], were significantly accumulated in *zmnac128 zmnac130* kernels. *OsNRAMP5*, which is an orthologous gene of *ZmNRAMP5* (Figure 4A), has been identified as the main Cd transporter in rice [44]. The expression of *ZmNRAMP5* was significantly upregulated in the endosperm filling of the *zmnac128 zmnac130* mutant compared to that in the WT (Figure 4C), indicating that this gene may be a major factor in the increased Cd content in the *zmnac128 zmnac130* kernels. However, DAP-seq analysis indicated that there were not any apparent peaks bound by ZmNAC128 and ZmNAC130 in the *ZmNRAMP5* promoter (Figure 4B). It is suggested that ZmNAC128 and ZmNAC130 seem to indirectly regulate the expression of *ZmNRAMP5*.

Moreover, the levels of two biofortified elements, Zn and Mg, significantly decreased in the mutant (Figure 3B). ZmNAC128 and ZmNAC130 were previously reported to control Zn accumulation in kernels by directly regulating *ZmYSL2* expression [26]. Hence, ZmNAC128 and ZmNAC130 have dual functions: they restrict the accumulation of toxic HMMs while promoting the biofortification of Zn and Mg in the kernel filling.

### 3.5. ZmNAC128 and ZmNAC130 Directly Regulate the Expression of Grain-Filling-Controlling P Transporter ZmPHO1;2

**Figure 5:**
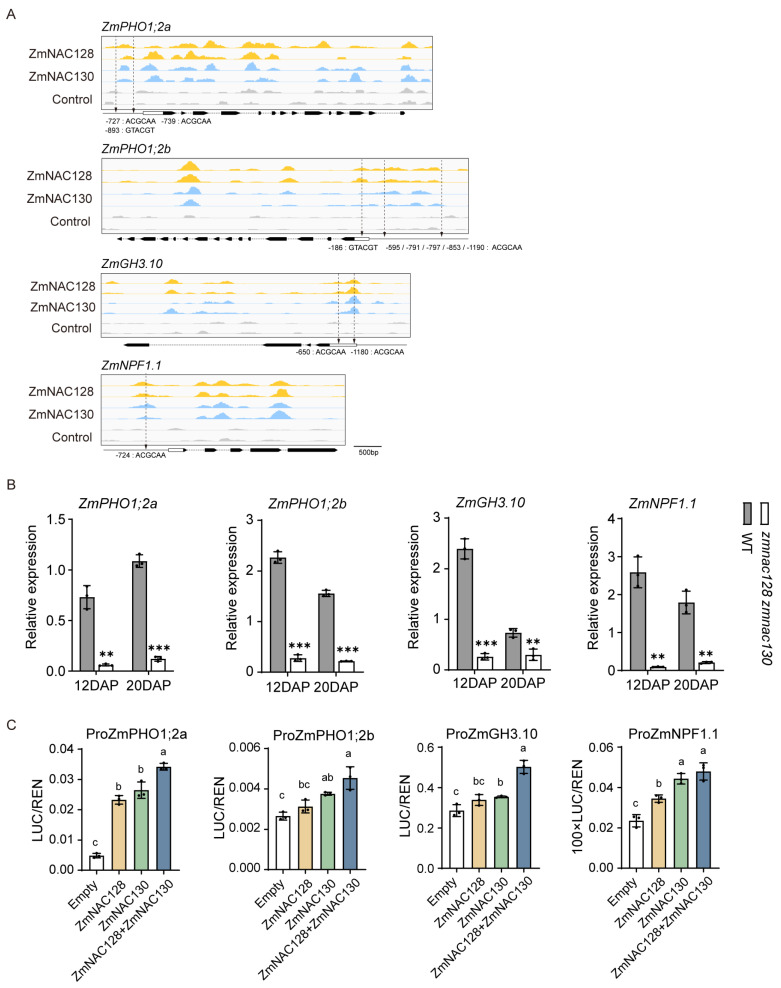
The identification of N-/P-related transporter genes and one JA-Ile synthesis-related gene as the direct targets of ZmNAC128 and ZmNAC130. (**A**) DAP-seq analysis shows the binding peaks of ZmNAC128 and ZmNAC130 on these four gene promoters. The cis-elements (‘ACGCAA’ and ‘GTACGT’), which were previously identified [25,26], are marked by arrows in the promoters. (**B**) A real-time PCR indicates the relative expression levels of these four genes in 12- and 20-DAP endosperms of the *zmnac128 zmnac130* mutant compared to those of the WT. *ZmActin* was used as a reference gene for normalization. (**C**) DLR assays detect LUC activities driven by the promoters in the presence of ZmNAC128, ZmNAC130, or both. Empty represents the negative control. The data represent the mean ± SD of 3 independent samples (**B**,**C**). Significant differences (** *p* < 0.01 and *** *p* < 0.001) were determined using a Student’s *t*-test (**B**). Different lowercase letters indicate significant differences according to a one-way ANOVA with Tukey’s multiple comparisons test (*p* < 0.05) (**C**).

Consistent with the significant reduction in P content (Figure 3C), two duplicate genes involved in maize grain phosphate transport, *ZmPHO1;2a* and *ZmPHO1;2b*, also exhibited significantly downregulated expression in the filing endosperms of the *zmnac128 zmnac130* mutant (Figure 5B; Table 1). Both *ZmPHO1;2a* and *ZmPHO1;2b* are highly expressed in endosperm filling (Figure S3) and are involved in determining the P content in kernels [8]. Electron in situ hybridization revealed that *ZmPHO1;2a* is expressed in the BETL, while *ZmPHO1;2b* is exclusively expressed in endosperm tissues surrounding the embryo (Figure S3). It is suggested that the two *ZmPHO1;2* genes function in the transfer of P at the interface of maternal/endosperm and endosperm/embryo.

To determine whether ZmNAC128 and ZmNAC130 directly regulate the expression of *ZmPHO1;2*, we performed a DAP-seq, which revealed that there were peaks bound by the two NACs in the promoters of *ZmPHO1;2a* and *ZmPHO1;2b*, with conserved NAC-binding motifs around the peaks (Figure 5A). Furthermore, DLR results indicated that LUC activity driven by the *ZmPHO1;2a* promoter was significantly greater in the presence of ZmNAC128 or ZmNAC130 than in the empty control. Moreover, the co-occurrence of two NACs further enhanced LUC activity compared to that of either one. In addition, LUC activity driven by the *ZmPHO1;2b* promoter was significantly increased in the presence of ZmNAC130 but not ZmNAC128 and the co-occurrence of the two NACs did not further enhance LUC activity compared to that of ZmNAC130 (Figure 5C). The results demonstrated that ZmNAC130 and ZmNAC128 play major roles in *ZmPHO1;2a* expression and minor roles in *ZmPHO1;2b* expression.

### 3.6. A Regulatory Mechanism of ZmNAC128 and ZmNAC130 in the Expression of the Endosperm-Specific Nitrate Transporter ZmNPF1.1

Among the downregulated transporter genes of these 243 hdEGs (Table 1), *ZmNPF1.1* is particularly interesting because it is a N transporter gene that is preferentially expressed in the BETL (Figure S3). A real-time PCR confirmed that the expression of *ZmNPF1.1* was significantly downregulated in the endosperm filling of the *zmnac128 zmnac130* mutant (Figure 5B). To investigate whether ZmNAC128 and ZmNAC130 directly regulate the expression of *ZmNPF1.1*, DAP-seq analysis was performed, and the results revealed that there were apparent peaks bound by ZmNAC128 and ZmNAC130 in the *ZmNPF1.1* promoter, with the presence of the conserved NAC-binding motif around the peaks (Figure 5A). DLR assays further indicated that, compared to that in the empty control, LUC activity driven by the *ZmNPF1.1* promoter was significantly greater in the presence of ZmNAC128 or ZmNAC130, but the co-occurrence of two NACs did not significantly enhance the LUC activity (Figure 5C).

Furthermore, a previous study revealed that an exogenous jasmonic acid (JA) treatment strongly induces the expression of *ZmNPF1.1* [45]. Interestingly, among these 243 hdEGs, *ZmGH3.10* (Zm00001eb338800), which belongs to Group I of the GH3 (Gretchen Hagen3) family and is associated with JA-amino acid conjugation [46,47], was downregulated. As the conjugation of the amino acid isoleucine (Ile) to JA is critical for conferring major bioactivity and is central to JA signaling and responses [46], we utilized an ultraperformance liquid chromatography/tandem mass spectrometry (UPLC-MS/MS) system to measure the contents of JA and JA-Ile in the 20-DAP kernels of the *zmnac128 zmnac130* mutant. The results showed a significant reduction in both the JA-Ile and JA contents in the *zmnac128 zmnac130* mutant compared to those in the WT (Figure S4A), and the ratio of JA-Ile to JA was also significantly decreased in the mutant (Figure S4B). Since *ZmGH3.10* is highly expressed in endosperm filling (Figure S3), it likely plays an important role in the JA-Ile synthesis in maize grains. DAP-seq analysis revealed the binding peaks of ZmNAC128 and ZmNAC130 in the promoter with conserved NAC-binding motifs around the peaks (Figure 5A). DLR further confirmed that, compared to that in the empty control, LUC activity driven by the *ZmGH3.10* promoter was significantly greater in the presence of ZmNAC130 but not in the presence of ZmNAC128, while the co-occurrence of the two NACs further increased the LUC activity compared to that in the case of ZmNAC130 (Figure 5C).

Taken together, these results indicate that ZmNAC128 and ZmNAC130 not only directly regulate the expression of *ZmNPF1.1*, but also control the synthesis of active JA-Ile by regulating the expression of *ZmGH3.10*. This, in turn, modulated the expression of *ZmNPF1.1* through JA signaling.

## 4. Discussion

It is now widely recognized that a TF can influence the expression of hundreds, or even thousands, of genes in the tissues where it is expressed and functions. However, only a limited number, typically a few or at most dozens, of downstream genes of a single TF have been identified. Understanding the core regulatory network of a TF is akin to solving a complex puzzle, necessitating continuous exploration of its downstream genes to complete the picture. For instance, the regulatory network of O2, a core TF involved in grain filling in maize, has been extensively studied for more than 30 years. Numerous studies have contributed to expanding the body of knowledge of O2 [14,15,16,17,26,27,48,49,50,51]. Therefore, exploring the core regulatory network of a TF is a complex and challenging endeavor.

The seed is one of the most important sink organs of plants; it not only absorbs and assimilates from mature plants but also transports various nutrients and toxic HMMs from underground root tissues. The transport of these nutrients or toxic HMMs across the maternal/filial interface of the seed is tightly regulated by a diverse array of transporters. Targeting these transporters could be a promising direction for crop improvement. A recent study on iron biofortification in maize kernels is enlightening, as it revealed that by manipulating the expression of *ZmNAC78*, researchers enhanced the expression of its downstream iron transporters in the BETL and thereby improved the iron content in kernels [52].

**Figure 6:**
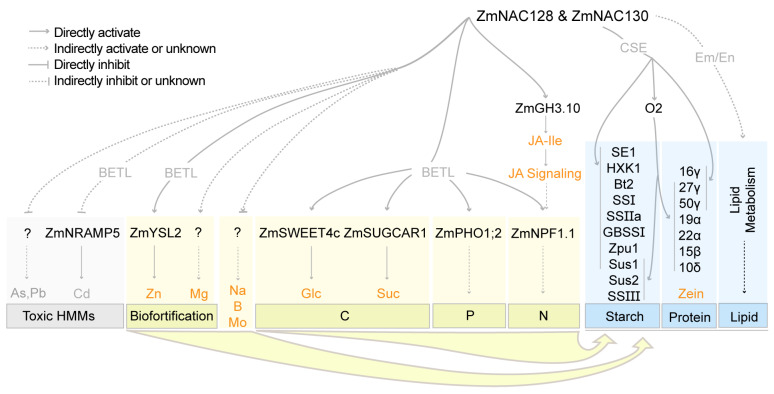
A working model for ZmNAC128 and ZmNAC130 acting as central coordinators in limiting toxic HMMs, promoting biofortification, and regulating the uptake and storage metabolism of C, N, and P nutrients. All target genes of ZmNAC128 and ZmNAC130 in this model were identified by the present study and our previous studies [25,26].

This study showed that ZmNAC128 and ZmNAC130, as versatile central coordinators, control the accumulation of numerous substances and the synthesis of three major storage compounds (starch, protein, and lipids) in maize grains (Figure 6). First, ZmNAC128 and ZmNAC130 inhibit the expression of many transporters (ZmNRAMP5, MATE genes, etc.) to limit the entry of toxic HMMs into kernels. Second, ZmNAC128 and ZmNAC130 enhance the biofortification of Zn and Mg. These genes directly regulate *ZmYSL2* expression and thus promote Zn uptake. Third, ZmNAC128 and ZmNAC130 control the uptake of C-, N-, and P-related nutrients by directly regulating the expression of five crucial transporters, *ZmSWEET4c*, *ZmSUGCAR1*, *ZmNPF1.1*, and *ZmPHO1;2*. These two NAC TFs positively influence the phosphorus content of seeds by regulating the expression of *ZmPHO1;2*. Natural phosphorus reserves are limited, highlighting the importance of developing phosphorus-efficient crops. The rice phosphorus-starvation tolerance 1 (*OsPSTOL1*) gene plays a crucial role in grain yield under phosphorus-deficient soil conditions [53]. The homologous gene (Zm00001d049727, *ZmPSTOL1*) of *OsPSTOL1* in maize is almost not expressed in developing kernels [6,54], suggesting that ZmNAC128 and ZmNAC130 are unlikely to directly regulate *ZmPSTOL1* expression. So, overexpression of *ZmPSTOL1*, *ZmNAC128*, and *ZmNAC130* in maize should be a potential strategy to significantly enhance the grain yield in phosphorus-deficient soil.

Moreover, ZmNAC128 and ZmNAC130 also regulate *ZmGH3.10* expression to control the active JA-Ile content and thus the JA signaling for *ZmNPF1.1* expression and grain filling. Combined with the uptake of macro- and micronutrients, the two NACs collaborate with O2 to regulate the expression of all types of zein genes and 10 starch metabolism genes, as well as lipid synthesis in kernels.

Based on these findings, we plan to further increase the expression of *ZmNAC128* and *ZmNAC130* via the use of a strong BETL-specific promoter. This strategy should simultaneously inhibit the accumulation of toxic HMMs and promote biofortification in kernels. To further facilitate the synthesis of seed storage compounds, we also plan to genetically combine *27-kD γ-zein* promoter-driving varieties with BETL-specific promoter-driving varieties. Ideally, the expression of *ZmNAC128* and *ZmNAC130* will be enhanced, especially in the BETL and CSE, without affecting other agronomic traits. Our ultimate goal is to breed maize varieties that exhibit high yield, superior quality, biofortification, and safety.

## Figures and Tables

**Table 1:** Information on the transporter genes in the 243 hdEGs.

Transporters		Putative Transport Function	Set-1		Set-2		Set-3	
Gene Name	Gene ID		log_2_(FC)	*p* Value	log_2_(FC)	*p* Value	log_2_(FC)	*p* Value
*ZmNRAMP5*	Zm00001eb304610	HMMs	3.26	1.4 × 10^−13^	1.31	3.1 × 10^−3^	3.62	7.9 × 10^−67^
*ZmMRPA14*	Zm00001eb072510		5.31	2.6 × 10^−68^	2.84	6.5 × 10^−11^	3.77	6.6 × 10^−73^
*ZmMATE11*	Zm00001eb048860		6.58	9.6 × 10^−144^	5.90	3.6 × 10^−26^	6.89	2.5 × 10^−267^
*ZmMATE17*	Zm00001eb086100		4.65	1.9 × 10^−57^	3.29	8.7 × 10^−6^	3.38	5.0 × 10^−115^
*ZmMATE30*	Zm00001eb006660		3.02	1.6 × 10^−52^	2.53	7.1 × 10^−22^	1.15	1.6 × 10^−4^
*ZmMATE48*	Zm00001eb405390		1.95	3.9 × 10^−14^	1.18	3.4 × 10^−3^	1.37	3.3 × 10^−24^
*ZmTIP1;1*	Zm00001eb003730	Water	2.18	2.7 × 10^−19^	1.69	8.7 × 10^−4^	1.45	1.1 × 10^−37^
*ZmSIP1-2*	Zm00001eb349840	Water	1.37	1.2 × 10^−6^	2.09	4.3 × 10^−8^	1.47	9.3 × 10^−48^
*ZmNCS2-2*	Zm00001eb042450	-	1.51	3.4 × 10^−16^	1.58	9.5 × 10^−10^	1.40	8.5 × 10^−66^
*ZmNPF1.1*	Zm00001eb152400	Oligopeptide, phosphate ion	−2.35	1.7 × 10^−30^	−2.26	2.1 × 10^−5^	−1.57	1.2 × 10^−14^
*ZmCOPT2*	Zm00001eb151980	Copper	−1.15	1.5 × 10^−6^	−2.20	4.8 × 10^−2^	−2.85	2.2 × 10^−38^
*ZmPIP2:2*	Zm00001eb096680	Water	−4.33	9.7 × 10^−6^	−3.34	3.9 × 10^−12^	−1.44	2.5 × 10^−32^
*ZmTIP3-1*	Zm00001eb221090	Water	−2.49	7.8 × 10^−4^	−1.91	8.1 × 10^−8^	−2.14	1.2 × 10^−34^
*cl34132_1a*	Zm00001eb068600	-	−2.12	1.3 × 10^−50^	−2.11	3.5 × 10^−12^	−1.13	1.8 × 10^−13^
putative transporter	Zm00001eb323920	Phosphate ion	−5.68	1.3 × 10^−37^	−6.09	9.1 × 10^−41^	−4.51	1.1 × 10^−133^
*ZmSWEET4c*	Zm00001eb236810	Glucose	−3.12	8.8 × 10^−10^	−2.56	2.7 × 10^−3^	−1.25	1.4 × 10^−34^
*ZmPHO1;2a*	Zm00001eb191650	Phosphate ion	−1.19	1.4 × 10^−16^	−1.58	5.8 × 10^−7^	−1.52	2.4 × 10^−17^
*ZmPHO1;2b* ^1^	Zm00001eb258520	Phosphate ion	−0.13	1.1 × 10^−2^	−1.09	3.78 × 10^−2^	−1.48	3.5 × 10^−37^

Note: ^1^ This study investigated *ZmPHO1;2b*, which is not among the 243 hdEGs. FC represents fold change. The *p* value was calculated with DESeq2R.

## Data Availability

RNA-Seq data are available from the National Center for Biotechnology Information Gene Expression Omnibus (http://www.ncbi.nlm.nih.gov/geo, accessed on 1 January 2024) under the accession number GSE252297.

## Supplementary Materials

The following supporting information can be downloaded at: https://www.mdpi.com/article/10.3390/genes15060663/s1, Figure S1: The lipid content was significantly reduced in the mature kernels of the *zmnac128 zmnac130* mutant; Figure S2: An electronic RNA in situ hybridization showing the spatial expression pattern of 18 transporter genes from Table 1 in 12-DAP kernels; Figure S3: The spatiotemporal expression pattern of *ZmPHO1;2*, *ZmNPF1.1*, and *ZmGH3.10*; Figure S4: The content and ratio of JA-Ile to JA in 20-DAP kernels of the *zmnac128 zmnac130* mutant; Table S1: The information of mapped reads in three sets of RNA-seq; Table S2: The proteins used as blastp query sequences; Table S3: The primer sequences used in this article; Dataset S1: The 243 hdEGs are commonly present in the three sets of transcriptomes from 16-DAP endosperms of zmnac128 zmnac130 mutants; Dataset S2: The raw data and detailed statistical analysis used in this study.
