# Supplementary material for: Central Roles of ZmNAC128 and ZmNAC130 in Nutrient Uptake and Storage during Maize Grain Filling
Source: Genes (Basel). 2024 May 23;15(6):663. doi: 10.3390/genes15060663 (PMC11203180; doi:10.3390/genes15060663)
Supplement: Supplementary file 1 [file genes-15-00663-s001.zip › genes-2975973-supplementary.pdf]

genes

MDPI

Supplementary figures and figure legends

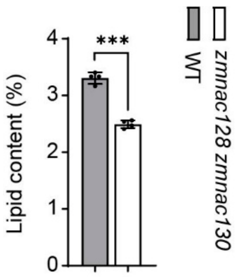

Figure S1. The lipid content was significantly reduced in mature kernels of the *zmnac128 zmnac130* mutant. The data represent the means ± SDs of 3-5 independent samples (A-H). Significant differences (***P < 0.001) were determined via Student's t- test.

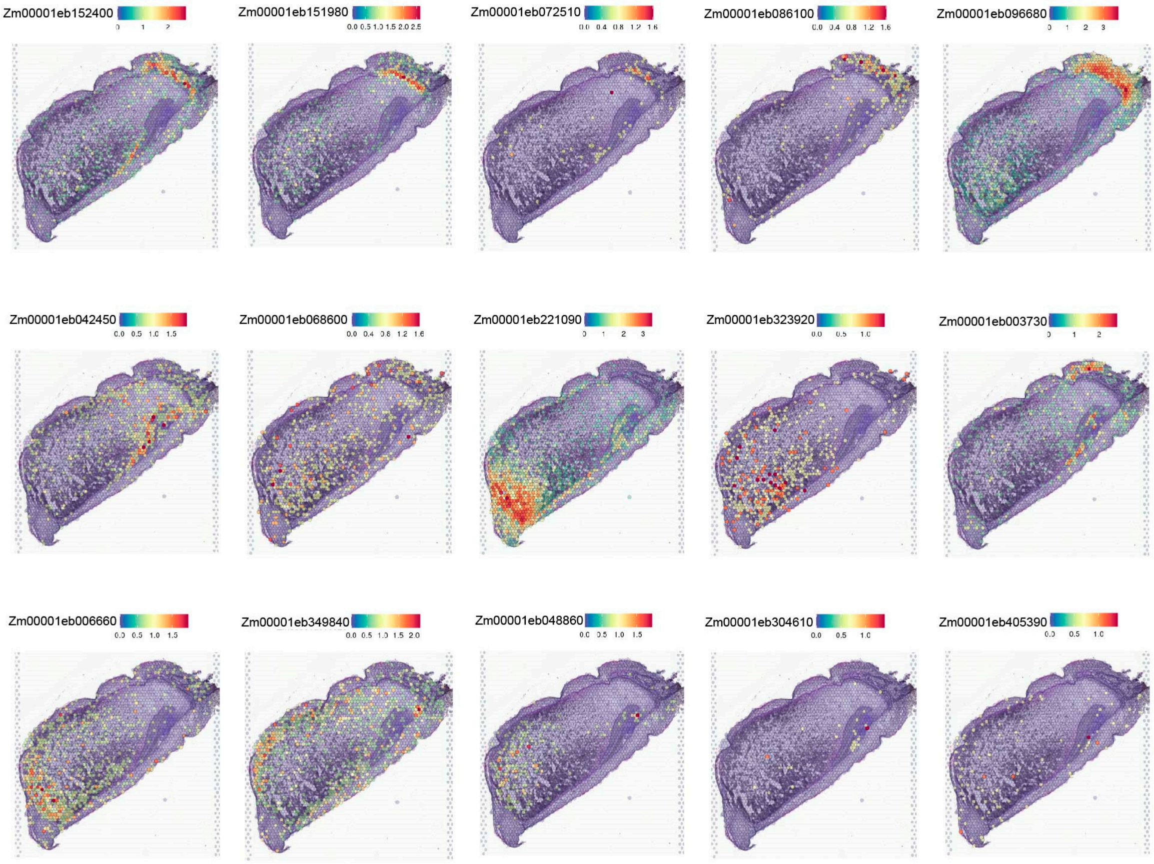

Figure S2. Electronical RNA in situ hybridization showing the spatial expression pattern of 18 transporter genes from Table 1 in 12-DAP kernels. The results were obtained from a web-based platform (http://119.78.67.206:3838) **[1]**.

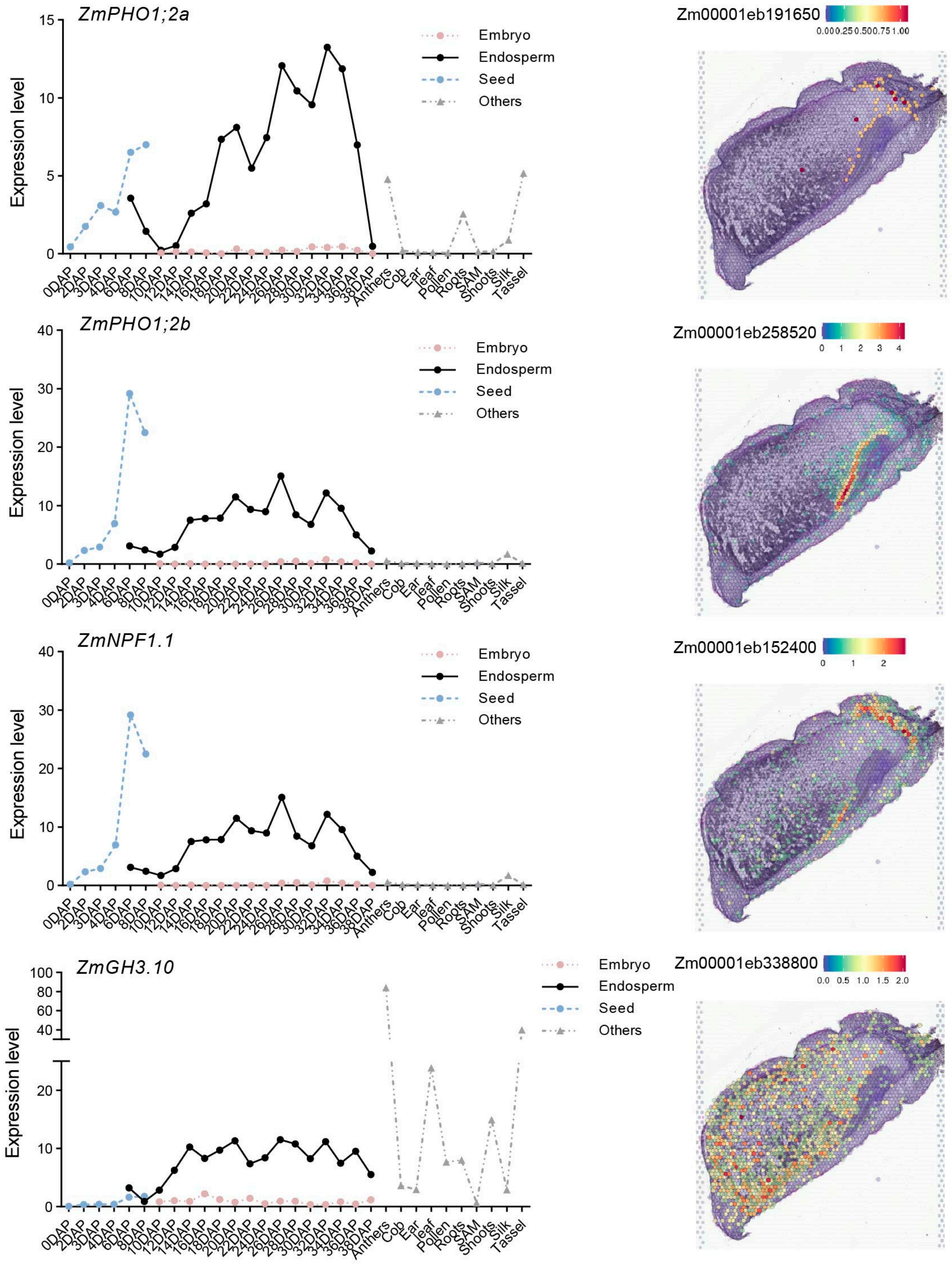

Figure S3. The spatiotemporal expression pattern of *ZmPHO1;2*, *ZmNPF1.1*, and *ZmGH3.10*. The data of temporal expression level was obtained from a previous study **[2]**, while the spatial expression was used the data of electronical in situ hybridization from a web-based platform (http://119.78.67.206:3838) **[1]**.

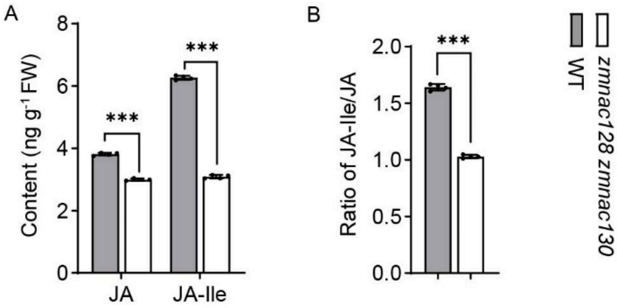

Figure S4. The content and ratio of JA-Ile to JA in 20-DAP kernels of the *zmnac128 zmnac130* mutant. The data represent the means ± SDs of 3-5 independent samples (A-H). Significant differences (***P < 0.001) were determined via Student's *t*- test.

Table S1. The information of mapped reads in three sets of RNA-seq.

Sample	Total clean reads	Average of total clean reads	Total Mapped(%)	Average of total mapped
Set-1-WT-1	54,823,284	60,195,153	49,826,079 (90.88%)	51,413,251 (91.42%)
Set-1-WT-2	49,662,578		45,263,339 (91.14%)	
Set-1-nac-1	66,108,152		60,725,809 (91.86%)	
Set-1-nac-2	54,254		49,837,776 (91.81%)	
Set-2-NT-1	41,402,634	43,145,919	39,358,845 (95.06%)	40,913,225 (94.82%)
Set-2-NT-2	41,865,216		39,858,443 (95.21%)	
Set-2-NT-3	45,752,282		43,164,651 (94.34%)	
Set-2-nac-1	47,935,558		45,605,130 (95.14%)	
Set-2-nac-2	41,094,082		38,943,591 (94.77%)	
Set-2-nac-3	40,825,746		38,548,687 (94.42%)	
Set-3-NT-1	41,432,552	42,374,768	38,462,100 (92.83%)	39,144,188 (92.40%)
Set-3-NT-2	38,172,099		35,473,672 (92.93%)	
Set-3-NT-3	42,681,527		39,584,841 (92.74%)	
Set-3-nac-1	47,026,895		42,986,500 (91.41%)	
Set-3-nac-2	43,240,158		39,945,709 (92.38%)	
Set-3-nac-3	41,695,378		38,412,311 (92.13%))	

Note: WT, wild type. NT, nontransgenic sibling segregated from crossing the inbred B73 with *zmnac128 zmnac130* double mutant in the inbred KN5585. nac, *zmnac128 zmnac130* mutant. -1, -2, and -3 represent three independent samples.

Table S2. Proteins used as BLASTP query sequences.

Identifier	Species	Source
AT1G15960.1	*Arabidopsis thaliana*	Phytozome 13
AT1G80830.1	*Arabidopsis thaliana*	Phytozome 13
LOC_Os07g15460.1	*Oryza sativa*	Phytozome 13
LOC_Os07g15370.1	*Oryza sativa*	Phytozome 13
LOC_Os01g53210.1	*Oryza sativa*	Phytozome 13
LOC_Os01g31870.1	*Oryza sativa*	Phytozome 13
LOC_Os03g41070.1	*Oryza sativa*	Phytozome 13
LOC_Os02g03900.1	*Oryza sativa*	Phytozome 13
LOC_Os06g46310.1	*Oryza sativa*	Phytozome 13
Zm00001eb304610_T004	*Zea mays*	Phytozome 13
Zm00001eb097120_T001	*Zea mays*	Phytozome 13
Zm00001eb113380_T002	*Zea mays*	Phytozome 13
Zm00001eb224770_T001	*Zea mays*	Phytozome 13
Zm00001eb231210_T001	*Zea mays*	Phytozome 13

Table S3. Primers used in this study.

			sequence (5' - 3')
DLR assay	SE1 promoter (pGREEN)	F	gtcgacggtatcgataagcttgattggaaaggaggaggtaggat
		R	ggcggccgctctagaactagtcgagagagcgctcctccg
	HXK1 promoter (pGREEN)	F	gtcgacggtatcgataagctttctatgtgtgtgggtgggtgg
		R	ggcggccgctctagaactagtgggaccgaacccccg
	ZmNPF1.1 promoter (pGREEN)	F	gtcgacggtatcgataagcttaagcgataacgacacttgtttcg
		R	ggcggccgctctagaactagtcgcttccgttcgctttcgag
	ZmPHO1;2a promoter (pGREEN)	F	gtcgacggtatcgataagcttacacccagacattgcgacc
		R	ggcggccgctctagaactagtcctccgcgaccgaccg
	ZmPHO1;2b promoter (pGREEN)	F	gtcgacggtatcgataagctttcttgctcgtgctcgcat
		R	ggcggccgctctagaactagtcgtgggatccgcctcg
	ZmGH3.10 promoter (pGREEN)	F	gtcgacggtatcgataagcttcttgtccctattccgggga
		R	ggcggccgctctagaactagtggcgtggcccgggcccggcc
	ZmNRAMP5 promoter (pGREEN)	F	gtcgacggtatcgataagcttccacctaggaaaggtcagacg
		R	ggcggccgctctagaactagtgcctagttgcctactctggg
	ZmNAC128 CDs (pRI101)	F	aaaacccgggatggcggaccagcagcagccacag
		R	aaaaggtacctcagtacttgtacttccatatgc
	ZmNAC130 CDs (pRI101)	F	aaaaggatccatggcggcggaccagcagccgcag
		R	aaaagaattctcagtacttccacacgccatccatc
RT-qPCR	SE1	F	gtcatgatgggggctatctga
		R	tggctctcgctgacgaaca
	HXK1	F	atttggcactggcacaaacg
		R	tgacctgaagtacagcaaaatcg
	ZmNRAMP5	F	aggcgcagcaagtctctaag
		R	cgtctccaagttgccaggat
	ZmPHO1;2a	F	ttgcgcaaggattggtgttc
		R	catctttcctgaccctggcg
	ZmPHO1;2b	F	ggaataagctcaggcgggtg
		R	tgcaacaattctcaatcgaccac
	ZmNPF1.1	F	ccggacgaaacagaaaagcc
		R	ccgaaccaaacgcaaatgct
	ZmGH3.10	F	ataccggacttcctacgcc
		R	atcgtgggcatgaactcctc
	ACTIN	F	gctacgagatgcctgatggtc
		R	cccccactgaggacaacg

References

[1] Fu Y, Xiao W, Tian L, et al. Spatial transcriptomics uncover sucrose post-phloem transport during maize kernel development. *Nature communications* **2023**, *14*, 7191-.

[2] Chen J, Zeng B, Zhang M, et al. Dynamic transcriptome landscape of maize embryo and endosperm development. *Plant Physiol* **2014**, *166*, 252-64.
